# Optimizing the role of pharmacists at the primary healthcare centres in Indonesia through an integrated information system

**DOI:** 10.3389/fpubh.2024.1446587

**Published:** 2025-01-27

**Authors:** Nur Yuliasih, Yasmin Fatinah, Rizky Abdulah, Auliya A. Suwantika

**Affiliations:** ^1^Faculty of Pharmacy, Department of Pharmacology and Clinical Pharmacy, Universitas Padjadjaran, Bandung, Indonesia; ^2^Center of Excellence in Higher Education for Pharmaceutical Care Innovation, Universitas Padjadjaran, Bandung, Indonesia; ^3^Center for Health Technology Assessment, Universitas Padjadjaran, Bandung, Indonesia

**Keywords:** desk review, qualitative study, semi-structured interview, health policy, analysis framework

## Abstract

**Introduction:**

The role of pharmacists at primary healthcare centers (PHCs) in Indonesia still needs long-term improvement. Enhancing interprofessional collaboration through data-driven collaboration is essential to strengthening cooperation. This study aimed to identify the need for an integrated information system to enhance the role of pharmacists at PHCs in Indonesia.

**Methods:**

A desk review was applied as the initial step to analyze the role of pharmacists at the PHCs in Indonesia. Furthermore, a qualitative study was conducted using Walt and Gilson’s health policy analysis framework. Semi-structured interviews were conducted in four sections (context, content, process, and actors) with consideration of selected key respondents. All interviews were transcribed verbatim and then analyzed using Braun and Clarke’s thematic analyses, ensuring a comprehensive understanding of the situation.

**Results:**

We identified two significant challenges in optimizing the use of integrated systems at the PHCs to increase the role of pharmacists. Considering context-content-process-actors, implementing integrated pharmaceutical care standards relies significantly on human resources and infrastructures. Key challenges related to human resources are a limited number of human resources, a high workload, and insufficient use of working tools. We found several challenges regarding infrastructure, such as network connectivity issues, non-integrated systems or applications, and suboptimal benefits from the current systems.

**Conclusion:**

Various systems or applications in PHC involve reporting to the Ministry of Health, but detailed integration of these systems needs to be achieved immediately. According to informants, the criteria for desired applications are crucial to optimizing the integrated system, using it, and streamlining tasks for pharmacists at PHCs. Common expectations include an integrated system for monitoring drug usage and orders. Apart from usefulness, network connectivity must be assured for accessibility by all parties.

## Introduction

As a geographically complex nation, Indonesia is one of the most populated countries, with a population of 278 million (1). Currently, the country faces the unique challenge of providing healthcare services across more than 1,000 inhabited islands with a combination of public and private providers (2). Since 1960, the government has introduced primary healthcare centers (PHCs) to provide better healthcare services through public providers (3). Over the next 60 years, the role of pharmacists at PHCs in Indonesia still needs to be improved. Maintaining service delivery, including clinical pharmacy service, through the PHCs network as a significant source of primary care remains challenging (4). By learning from the experience of other countries, an integrated information system at PHCs is crucial to provide clear documentation and appropriate information in making decisions related to pharmacy practice. In particular, an adequate health information system can be used as a basis for decision-making processes and for establishing evidence-based policies to improve patients’ safety.

The PHCs in Indonesia are targeted as health service facilities that organize public health efforts by utilizing the role of healthcare professionals. Evidence is growing that healthcare services delivered by collaborative practitioners are superior to a single practitioner (5). Improving interprofessional collaboration through data-driven collaboration is essential to strengthening cooperation (6). Hence, a comprehensive study is required to explore the enhanced role of pharmacists at the PHCs that can be optimized through an integrated information system. As an initial step, a qualitative study collecting data from targeted key respondents is necessary to identify the need for an integrated information system to enhance the pharmacist’s role.

## Methods

**Figure 1:**
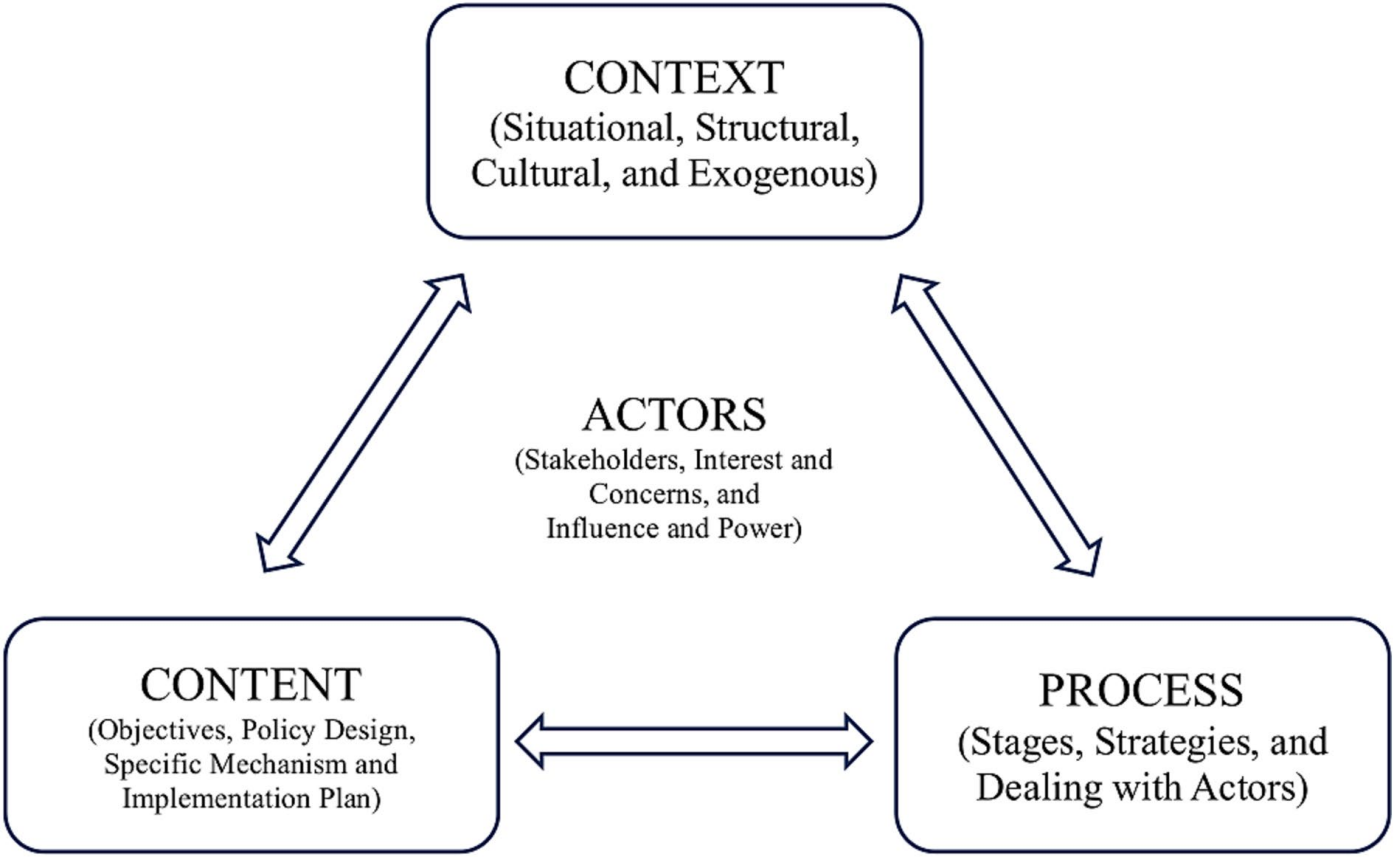
Framework for health policy analysis.

A review of available data, existing policy, legal review, and published literature was applied as the initial step to analyze the role of pharmacists at the PHCs in Indonesia. The desk review aimed to identify the current problem and their potential exit ways. Furthermore, a qualitative study was conducted using Walt and Gilson’s health policy analysis framework (see Figure 1) (7).

As a pilot study of an integrated information system at the PHCs in Indonesia, we focused on Tegal, a district in Central Java Province with 31 PHCs. According to the current regulation, each PHC in Indonesia must have a pharmacist to provide pharmaceutical services (8). In the case of a PHC that does not have a pharmacist, the provision of limited pharmaceutical services is carried out by technical personnel under the supervision of a pharmacist appointed by the District of Health (8). Not many districts in Indonesia have pharmacists in each PHC, and Tegal is one of them. Selecting this district as a reference case was expected to represent the broader PHC landscape in the optimal situation of providing pharmaceutical services managed by pharmacists.

A qualitative study with semi-structured interviews was conducted, considering selected key respondents (*n* = 31 pharmacists from 31 PHCs). The primary inclusion criteria for the respondent selection were pharmacists at the PHCs willing to be involved since, in the study, we only focused on optimizing the role of pharmacists at PHCs through an integrated information system from their perspectives in running two major daily activities: pharmaceutical management and clinical activities. Both activities were explored during the interviews and delivered in four sections: context, content, process, and actors. More detailed information about the list of questions in each section can be seen in Table 1. All interviews were transcribed verbatim and then analyzed using Braun and Clarke’s thematic analyses, ensuring a comprehensive understanding of the situation inductively (9).

**Table 1:** List of questions for semi-structured interviews.

No.	Questions
Context	
1.	What is your opinion about the implementation of Ministry of Health regulation No. 26/2020 concerning Standards for Pharmaceutical Services at the PHC?
2.	What do you think about the supporting facilities available at the PHC?
3.	What do you think about the existing system or application to support pharmaceutical services at the PHC?
4.	Do you think that the existing system or application at the PHC is connected to other systems or applications?
Content	
5.	What are the major criteria of system or application to be applied in optimizing the pharmaceutical services at the PHC?
6.	How does the existing system or application assist in the flow of pharmaceutical supply chain management?
7.	How does the existing system or application assist in prescription services and other clinical pharmacy services?
8.	What challenges and obstacles are encountered in using the existing system or application at the PHC?
Process	
9.	How many systems or applications are currently being used to support pharmaceutical services at the PHC? What are the advantages and disadvantages of using these systems or applications?
10.	How is your experience in using the existing system or application to support pharmaceutical services at the PHC?
11.	What type of pharmaceutical services reporting at the PHC must be documented regularly? What is the existing system or application used for reporting?
12.	What type of the existing system or application do you find easy and helpful in carrying out pharmaceutical service activities at the PHC?
Actors	
13.	What do you think about the support from the local government (e.g., District of Health) and the central government (e.g., Ministry of Health) for the implementation of pharmaceutical services at the PHC?
14.	Who is responsible for data entry in the existing system or application to support pharmaceutical services at the PHC?
15.	What can be improved from the existing system or application to optimize the pharmaceutical services at the PHC? Who should be responsible with this?

## Results

**Figure 2:**
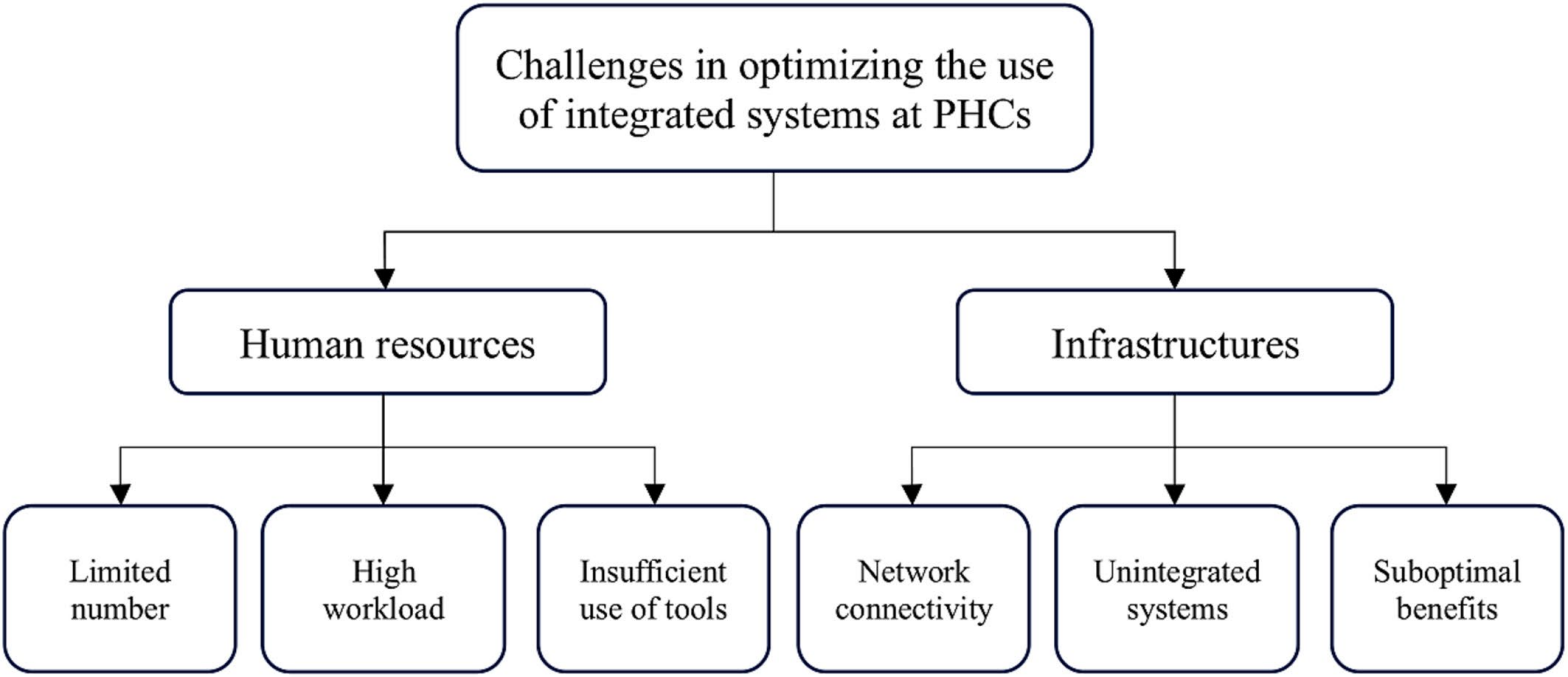
Challenges in optimizing the use of integrated systems.

Our desk review focused on relevant documents and identified two significant challenges in optimizing the use of integrated systems at the PHCs to increase the role of pharmacists. Considering the current situation, the implementation of pharmaceutical care standards relies significantly on human resources and infrastructures. Key challenges related to human resources are a limited number of human resources, a high workload, and insufficient use of working tools. We found several challenges regarding infrastructure, such as network connectivity issues, non-integrated systems or applications, and suboptimal benefits from the current systems (see Figure 2).

Considering the policy framework, the transcription process involved coding and categorization into each category according to the framework, as illustrated in Figure 2. This methodology has allowed us to analyze and present our findings systematically (Table 2).

**Table 2:** Coding results using the policy framework.

Coding results	Descriptions	Policy framework
Inadequate infrastructure	Challenges in using the application	Context
Insufficient human resources		
The application is not yet optimally utilized		
Suboptimal implementation of pharmaceutical service standards	Unintegrated applications or systems	Content
Lack of integration in the pharmaceutical care workflow		
Numerous items and menu options pose challenges in data entry	Barriers and criteria required in using the application	Process
The need for an integrated application from prescription to service		
Application usage is carried out by pharmacist in each PHC	Optimizing the use of application	Actors
Efforts to support application usage		

### Context

Informants detailed the main difficulties in implementing regulations of PHCs, which revolve around limited human resources. According to the regulation issued by the Minister of Health number 74 of 2016, the pharmacist-to-patient ratio is set at 1:50 per day for each PHC (8). Other informants also provided insights, mentioning that pharmacists may perform tasks beyond their responsibilities, leading to suboptimal performance. One informant explained:

*“The actual implementation of pharmaceutical services in our PHC is not fully in line; only some things run smoothly, primarily due to Human Resources (HR) constraints. Only one person in the HR is assisted by other professions, resulting in suboptimal adherence to service standards* [P10]*.”*

In implementing pharmaceutical services, meeting the requirements for facilities and infrastructure poses a common challenge in various PHCs despite differences in available facilities. Several informants highlighted deficiencies in service and drug storage areas, affecting the availability and provision of medications. Some explanations include:

*“Regarding facilities at our PHC, they do not meet standards, especially in service rooms and drug storage; they do not comply with the standards. Requesting drugs often takes time and is neglected, resulting in not all medications being dispensed* [P2, P5, P21]*.”*

Another informant added that a significant hurdle related to facilities and infrastructure is the availability of refrigerators to support cold chain storage, as mandated by regulations. The informant elaborated on the storage conditions:

*“Regarding facilities and infrastructure at my PHC, there is no refrigerator. So far, drugs that require storage in a refrigerator are entrusted to the laboratory for safekeeping* [P13]*.”*

Using systems or applications to support pharmaceutical services in PHC is considered reasonably beneficial; however, several informants highlighted challenges in application usage, such as the extensive menu options leading to prolonged data input. One informant expressed concerns about the impact of these challenges on the suboptimal implementation of the system:

*“So far, at the health center, we have been supported by an application from the health office, but one of the challenges is that the data in that application needs to be integrated. Many items need to be input, making the service time-consuming. Some clinics are reluctant to input data due to the abundance of items, making it inefficient. Thus, even though we have a system, we have not fully implemented it* [P29]*.”*

### Content

According to informants, the criteria for desired applications are crucial to optimize the application, use, and streamline tasks for pharmacists at PHCs. Common expectations include an integrated system for monitoring drug usage and orders. One informant, emphasizing the importance of the pharmacists’ role in this process, explained:

*“The criteria for an application should be understandable, with not too many items/features. One application is expected to be integrated into other reports. The number of menu options is also crucial to keep it as simple as possible for efficiency* [P24]*.”*

Another participant’s response indicated that the current systems or applications do not optimally support prescription and other pharmaceutical services. This situation is related to time-consuming data entry processes, impeding services to other patients. The impact of these challenges is explained as follows:

*“For the prescription service flow, from receiving the prescription to delivering the medication to the patient, we have been using the available application. The time-consuming data entry makes it difficult, so we often report things manually* [P31].*”*

### Process

According to several informants, the current application would be beneficial if adequate personnel could process data entry quickly. Manual reporting remains the preferred option due to the challenges faced. Various systems or applications in PHC involve reporting to the Ministry of Health, but detailed integration of these systems is yet to be achieved. Additionally, some informants express difficulties learning to use these applications and the time required for data entry. One informant provides detailed insight:

*“The application has a menu for medications and several other menus. Learning the many ways to utilize the application for optimal use takes time. Moreover, adding drug items to the application requires confirmation from the health office, contributing to the time needed* [P7]*.”*

The frequency of drug usage reporting supports another additional need related to an integrated information system. This reporting is predominantly done manually, as explained by an informant:

*“The need for documentation of drug usage reports as part of pharmaceutical care should be done every month, but most of this documentation is still carried out manually* [P19]*.”*

### Actors

Informants express varying opinions regarding the support from the central government and PHC leadership in organizing pharmaceutical service activities. While some informants acknowledge good support from the central government and PHC leadership, others point out instances where PHC leaders may need to adequately facilitate pharmacist input in meeting the minimum standards for infrastructure and human resources in pharmaceutical services. In this context, only pharmacists have the authority to report drug usage. One informant explains:

*“The application is under the responsibility of the pharmacist. Interpreting it for the HIV & AIDS information system and reporting vaccine stock requires IT personnel, and only pharmacists can make such reports* [P24]*.”*

According to informants, improvements to the current system/application include facilitating all activities, from registration processes to documentation in the pharmacy space. Collaboration between system/application developers and users is expected to make the application more efficient and effective, including the report from PHCs to the District of Health and the Ministry of Health. Additionally, ensuring sufficient internet connectivity is a primary focus for enhancement. Apart from usefulness, network connectivity must be assured for accessibility by all parties. One informant suggests a collaboration system:

*“The application should collaborate with the pharmacy warehouse to operate it with integration between availability, ordering, and reporting accessible in the same application for mutual benefit* [P20]*.”*

## Discussion

Community pharmacy practice in low-and middle-income countries, including Indonesia, is often described as in the state of infancy with several intractable barriers that have been substantially and continuously hampering the practice (10). Such a description might be valid in highlighting how pharmacy is practiced and the conditions within and beyond community pharmacy organizations. However, it is essential to recognize the potential benefits of integrating community pharmacy into the primary care system. Even though community pharmacies have been operating within communities for years, this concept is outside the contemporary discourse. However, in the case of Indonesia, we argue that changes in the healthcare system within the past decade, particularly with the introduction of universal health coverage (UHC) in 2014, have significantly amplified the role of pharmacists (11).

Pharmaceutical service activities at PHCs in Indonesia are a key component in improving the quality of health services for the community (12). According to regulations, these services are divided into two categories: pharmaceutical preparation management activities and clinical pharmaceutical service activities, both of which are supported by human resources and infrastructure (8). The role of pharmacists in these activities is crucial. They are responsible for managing pharmaceutical supplies and services, requiring efficiency and effectiveness. One of the ways to achieve this is through the use of systems or applications. However, in practice, documentation is carried out manually, and it is not yet an option to use it in a systematic and integrated manner.

The current number of pharmacists working in PHCs is about 13,279, and the actual need is about 12,155 (13). Next to this significant gap, another challenge is the distribution. Like other healthcare professionals, the distribution of pharmacists remains centralized in Java, the most populous island in Indonesia. Considering the limited number of pharmacists and their primary responsibilities in PHCs, the current main activity of pharmacists in PHCs is more focused on pharmaceutical preparation management than clinical pharmaceutical service activities. Hopefully, using an integrated information system can distribute this workload in a more balanced way. This effort is linear with the Ministry of Health’s roadmap, highlighting that the ideal ratio of pharmacists in PHCs to deliver daily clinical pharmaceutical service is one pharmacist to cover fifty patients (8).

From the Ministry of Health’s perspective, budget is not a significant constraint to improving the number of healthcare professionals, including pharmacists. The major cause of the shortage of pharmacists in Indonesia is the limited number of professional pharmacist study programs. This program is only 22% of the total undergraduate pharmacy study programs (13). However, dealing with this bottleneck requires severe commitment and long-term improvement. The need to accelerate the implementation of an integrated information system in PHCs is very evident.

The World Health Report demonstrates how critical PHC is to changing global health systems. Lessons can be drawn from the experiences of Canada, Brazil, and Thailand, which have successfully accelerated the PHC reform phase. They concentrated on three things to make progress in the productive discussion of relevant policies: (i) the significant revolution of the Health Information System (HIS), (ii) the systematic utilization of innovations, and (iii) the sharing of knowledge and resources with multiple stakeholders. In particular, the report emphasizes the function of HIS as information providers needed to inform health policy and, more generally, to satisfy the demands of various users and organizations (14).

According to health system regulations in Indonesia, the HIS is a set of arrangements supported by interconnected data, information, indicators, procedures, devices, technology, and human resources (15). Integrated healthcare services, grounded in robust primary care and public health roles, play a direct role in improving the distribution of health outcomes and enhancing overall well-being and quality of life. This setting, in turn, leads to significant economic, social, and individual advantages. Integrated care is associated with enhanced service accessibility, reduced unnecessary hospitalizations and readmissions, improved adherence to treatment, heightened patient satisfaction, increased health literacy and self-care, greater job satisfaction among healthcare professionals, and an overall enhancement in health outcomes (16).

Using the United States of America’s pharmacy information system implementation as another example, the government developed the systems to meet the corresponding workload and technology demands and to provide evidence-based medical treatment. The automation of pharmacy processes, including verification of patient identity and automated medication alerts, when linked to other advanced clinical information systems such as computerized physician order entry systems and the electronic medical record, has a synergistic effect on improving healthcare quality. Therefore, their growing investment in pharmacy information systems may offer cutting-edge pharmaceutical support, improving patient safety and the standard of healthcare (17).

Our results highlighted two significant challenges in using integrated systems at PHCs to optimize pharmacists’ roles. Considering the current situation, the implementation of pharmaceutical care standards relies significantly on human resources and infrastructure. Key challenges related to human resources that can be identified are a limited number of human resources, high workload, and insufficient use of working tools. We found several challenges regarding infrastructure, such as network connectivity issues, non-integrated systems or applications, and suboptimal benefits from the current systems.

The agenda for universal health coverage in Indonesia has spurred the exploration of various innovative approaches to expanding health services, including pharmaceutical services, to the general population (18). As the Ministry of Health has integrated digital health tools into its strategic approach to expanding health services, there is a pressing need to establish a standard framework for pharmaceutical services nationwide. Accelerating this effort, the Ministry of Health should consider three major priorities: identifying key challenges, learning best practices, and making evidence-informed policy. Learning from implementing digital solutions for PHC workers in Africa, a study by Owoyemi et al. (19) highlighted the significant challenges faced, such as limited coverage and network connectivity, lack of technological competence, lack of power supply, limited mobile phone usage, and application design challenges. Focusing on implementing integrated digital healthcare in primary care, Rodriguez et al. (20) emphasized limited digital literacy as a barrier to the integration system at the PHCs, which could lead to inequity. These challenges are pertinent to the current situation in Indonesia, as evidenced by our study.

Using the accelerated efforts to improve PHCs’ services during the COVID-19 pandemic through digitalization and its integration as an example, a study by Silva et al. (21) demonstrated the positive and negative impacts of not fully utilizing the potential of technologies. This situation underscored the disparities in the rapid and urgent integration of digital tools in PHCs worldwide. Using this reference case as another priority, the government of Indonesia must bolster PHCs’ response capacity and expand the use of information and communication technologies using scientific evidence. Integrating digital health into public service is paramount, particularly in the COVID-19 pandemic, which has catalyzed fundamental shifts across the healthcare delivery system, including PHCs (22). While accelerated technology offers numerous potential advantages, not all healthcare providers and patients are equally prepared to participate in this intervention, which could pose concerns for health equity during and after the pandemic.

Our study is the first qualitative study in Indonesia to identify the need for an integrated information system at PHCs. Nevertheless, it has several limitations, and one of the significant limitations is the setting of the study. We focused this initial study on Tegal, a district in Central Java Province where all PHCs have pharmacists. Using this region as the case study, we expect the results of this study to be one of the references to enhance the pharmacist’s role through an optimal digitalization system. Further study is necessary to understand the indicators and components of the system in detail and consider user accessibility as an effort in system development. Key points can be drawn from the initial identification through this study for further development.

## Conclusion

Various systems or applications in PHCs involve reporting to the Ministry of Health, but detailed integration of these systems needs to be achieved immediately. According to informants, the criteria for desired applications are crucial to optimizing the integrated system, using it, and streamlining tasks for pharmacists at PHCs. Common expectations include an integrated system for monitoring drug usage and orders. Apart from usefulness, network connectivity must be assured for accessibility by all parties.

## Data Availability

The original contributions presented in the study are included in the article/supplementary material, further inquiries can be directed to the corresponding author.
